# Formulating multi diseases dataset for identifying, triaging and prioritizing patients to multi medical emergency levels: Simulated dataset accompanied with codes

**DOI:** 10.1016/j.dib.2020.106576

**Published:** 2020-12-06

**Authors:** Omar H. Salman, Mohammed I. Aal-Nouman, Zahraa K. Taha, Muntadher Q. Alsabah, Yaseein S. Hussein, Zahraa A. Abdelkareem

**Affiliations:** a Networking Department, Faculty of Engineering, AL Iraqia University, Baghdad, Iraq; b College of Information Engineering, Al-Nahrain University, Baghdad, Iraq; c Department of Electronic and Electrical Engineering, University of Sheffield, Sheffield S1 4ET, United Kingdom; d Information Systems and Computer Science Department, Ahmed Bin Mohammed Military College (ABMMC), P.O. Box: 22988, Doha, Qatar; e Alimam Aladham University College, Iraq

**Keywords:** E- health, Telemedicine, Healthcare, Sensor, Machine learning, ECG, Blood pressure, SpO2

## Abstract

This paper provides simulated datasets for triaging and prioritizing patients that are essentially required to support multi emergency levels. To this end, four types of input signals are presented, namely, electrocardiogram (ECG), blood pressure, and oxygen saturation (SpO2), where the latter is text. To obtain the aforementioned signals, the PhysioNet online library [1], is used, which is considered as one of the most reliable and relevant libraries in the healthcare services and bioinformatics sciences. In particular, this library contains collections of several databases and signals, where some of these signals are related to ECG, blood pressure, and SpO2 sensor. The simulated datasets, which are accompanied by codes, are presented in this paper. The contributions of our work, which are related to the presented dataset, can be summarized as follow. (1) The presented dataset is considered as an essential feature that is extracted from the signal records. Specifically, the dataset includes medical vital features such as: QRS width; ST elevation; peaks number; cycle interval from ECG signal; SpO2 level from SpO2 signal; high blood (systolic) pressure value; and low-pressure (diastolic) value from blood pressure signal. These essential features have been extracted based on our machine learning algorithms. In addition, new medical features are added based on medical doctors' recommendations, which are given as text-inputs, e.g., chest pain, shortness of breath, palpitation, and whether the patient at rest or not. All these features are considered to be significant symptoms for many diseases such as: heart attack or stroke; sleep apnea; heart failure; arrhythmia; and blood pressure chronic diseases. (2) The formulated dataset is considered in the doctor diagnostic procedures for identifying the patients' emergency level. (3) In the PhysioNet online library [1], the ECG, blood pressure, and SpO2 have been represented as signals. In contrast, we use some signal processing techniques to re-present the dataset by numeric values, which enable us to extract the essential features of the dataset in Excel sheet representations. (4) The dataset is re-organized and re-formatted to be presented in a useful structure feasible format. Specifically, the dataset is re-presented in terms of tables to illustrate the patient's profile and the type of diseases. (5) The presented dataset is utilized in the evaluation of medical monitoring and healthcare provisioning systems [2]. (6) Some simulated codes for feature extractions are also provided in this paper.

## Specifications Table

**Table:** 

Subject	Emergency medicine
Specific subject area	Triaging, classifying, prioritizing patients, medical services in emergency departments (Eds), remote patients in telemedicine, and E-health monitoring systems.
Type of data	Excel spread sheets, table.
How data has been obtained	The ECG, blood pressure, and SpO2 signals have been collected and downloaded from the online library Physionet [1]. We have applied some signal processing algorithms in order to extract the essential features of the datasets. The algorithms have been implemented and simulated in a real-time software environment. The outcomes of the simulated data are organized, structured, formulated and presented as multi diseases dataset.
Data format	Raw and analysed.
Parameters used in the data collection	Parameters include medical essential features such as: QRS width; ST elevation; Peaks number and cycle interval from ECG signal; SpO2 level from SpO2 signal; high Blood (*systolic*) pressure value; and low-pressure (*diastolic*) value from blood pressure signal. In addition, text-inputs parameters are collected as input data, which are represented by chest pain, shortness of breath, palpitation and whether patient at rest or not.
Description of the collected data Simulation Setup	The ECG, blood pressure, and SpO2 signals have been collected from the online library Physionet [1]. Each signal has more than 2000 elements. Each element in the signal has two values. The first value represents the time and the second represents voltage. The array of each signal has two columns where each column is represented by a value. The number of rows is defined by the number of elements in the signal, which starts from (0) and it ends at (n). A real time data processing algorithm is used to extract the required features. We have implemented our algorithms in different simulation environments. The proposed algorithms are implemented using the JAVA software programming language. Moreover, cross-platform, Apache, MySQL, PHP, and Perl, known as (XAMMP), is used in this paper. Based on our simulation, the dataset is re-organized and re-formatted to be presented in a structure dataset format. In addition, the dataset is re-presented using tables. The dataset illustrates the patient's profile and the type of diseases.
Data source location	We describe simulated data accompanied by codes. However, the required information for the database sources is provided in PhysioNet, which is a repository of freely available medical research data that is managed by the Massachusetts Institute of Technology (MIT) Laboratory for computational physiology [1]. City: Boston Country: United States of America (USA) Laboratory for computational physiology MIT, E25–505 45 Carleton St. Cambridge, MA 02139
Data accessibility	The primary data sources are available in a public repository and given in [1] A. L. Goldberger, L. A. N. Amaral, L. Glass, J. M. Hausdorff, P. Ch, R. G. Mark, J. E. Mietus, G. B. Moody, C. K. Peng, H. E. Stanley, and P. C. Ivanov, “PhysioBank, PhysioToolkit, and PhysioNet: Component of a New Research Resource for Complex Physiologic Signals,” Circulation, vol. 101, pp. E215–20, 2000, Direct URL to data: https://physionet.org/ Simulated data accompanied with codes are presented in https://data.mendeley.com/datasets/22d2kcr2yp/1
Related research article	[2] O. H. Salman, M. Aal-Nouman, Z. K. Taha, Reducing Waiting Time for Remote Patients in Telemedicine with Considering Treated Patients in Emergency Department Based on Body Sensors Technologies and Hybrid Computational Algorithms: Toward Scalable and Efficient Real Time Healthcare Monitoring System. DOI: 10.1016/j.jbi.2020.103592

## Value of the Data

The effectiveness of the presented dataset can be summarized as follows:

• The presented dataset contains some of the essential features of patients. In particular, these patients' features can be considered as significant symptoms indicators of many diseases such as: (a) Heart attack or stroke; (b) Sleep apnea; (d) Heart failure; (e) Arrhythmia; and (f) Blood pressure chronic diseases [3,4]. In addition, from the doctor diagnostics procedure point of view, these features can be considered as essential indicators of other sicknesses such as: (a) Adrenal gland tumours; (b) Thyroid problem; (c) Dementia; (d) Kidney disease; and (e) Peripheral arterial disease.
• The presented dataset is very beneficial to the researchers in both academic and industrial sectors. In particular, the presented dataset would allow researchers to make a fast decision and reliable assessment for the patients at the emergency level [7]. Specifically, such kind of assessment can be effectively used to decide whether the patients need fast medical services or can wait for a certain period and then served. As such, healthcare institutes, e.g., hospitals, would be able to provide fast and more efficient healthcare, and thus, increase their productive services and manage their medical resources more effectively.
• The presented dataset involves heterogeneous sources, which contain some medical sensors. i.e., ECG, SpO2 and blood pressure and text-inputs [5,7]. Such a combination of datasets provides valuable insights to the researchers in both academic and industrial business sectors. This particularly allows the researchers to design smart and intelligent healthcare systems, which are essential for the currently deployed Internet of Things (IoT) applications [8].
  The presented dataset is useful to a variety of research studies. For example, it can be used in triaging, classifying and prioritizing patients to multi emergency levels such as Risk, Urgent, Sick, Cold case and Normal.

## 1 Dataset description

**Fig. 1:**
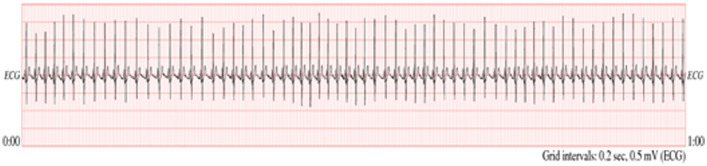
ECG Sample dataset for record a01 [1].
▪ Table 2 shows the description of SpO2 database and signal records. In addition, SpO2 signal sample is showed in Fig. 2.

The dataset presented in this paper includes ECG, blood pressure and SpO2 records and text-inputs. The dataset has been collected from PhysioNet databases [1]. However, the collected dataset is simulated, re-organized, re-structured in tables context to extract (1) some essential features from the signals, (2) database type, (3) signal record, (5) type of disease and (6) patients' profiles. All these details are presented in the attached appendixes with the following brief descriptions:

▪ Table 1 outlines the description of ECG databases and signals records along with all the patients' profiles. Moreover, a sample of the ECG signal is presented in Fig. 1.

**Table 1:** ECG databases and records that used in simulating algorithms.

Database	Record (Signal)	Record Description
Apnea-ECG data base (apnea-ecg)	a01	Male. Age: 51. Height: 175 (cm). Weight: 102 (Kg). 60 seconds (1 minute) length. Data standard format.

Apnea-ECG data base (apnea-ecg)	a03	Male. Age: 54. Height: 168 (cm). Weight: 80 (Kg). 60 seconds (1 min) length. Data standard format.

Apnea-ECG data base (apnea-ecg)	b01	Female. Age: 44. Height: 170 (cm). Weight: 63 (Kg). 60 seconds (1 min) length. Data standard format.

Apnea-ECG data base (apnea-ecg)	x15	Male. Age: 63. Height: 179 (cm). Weight: 104 (Kg). 60 seconds (1 min) length. Data standard format.

MIT-BIH Arrhythmia database (mitdb)	In most records, the upper signal is a modified limb lead II (MLII), obtained by placing the electrodes on the chest. The lower signal is usually a modified lead V1 (occasionally V2 or V5, and in one instance V4); as for the upper signal, the electrodes are also placed on the chest. This configuration is routinely used by the BIH Arrhythmia Laboratory. Normal QRS complexes are usually prominent in the upper signal.	

MIT-BIH Arrhythmia database (mitdb)	100	Male. Age: 69. 60 seconds (1 min) length. Signal (V5). Data standard format. The patient uses these Medications: Aldomet and Indera.

MIT-BIH Arrhythmia database (mitdb)	101	Female. Age: 75. 60 seconds (1 min) length. Signal (MLII). Data standard format. The patient uses this Medication: Diapres.

MIT-BIH Arrhythmia database (mitdb)	102	Female. Age: 84. 60 seconds (1 min) length. Signal (V5). Data standard format. The patient uses this Medication: Digoxin

MIT-BIH Arrhythmia database (mitdb)	103	Male. Age: Not Recorded. 60 seconds (1 min) length. Signal (V5). Data standard format. The patient uses these Medications: Diapres and Xyloprim.
MIT-BIH Arrhythmia database (mitdb)	105	Female. Age: 73. 60 seconds (1 min) length. Signal (MLII). Data standard format. The patient uses these Medications: Digoxin, Nitropaste and Pronestyl.

MIT-BIH Arrhythmia database (mitdb)	106	Female. Age: 24. 60 seconds (1 min) length. Signal (MLII). Data standard format. The patient uses this Medication: Inderal.

MIT-BIH Arrhythmia database (mitdb)	107	Male. Age: 63. 60 seconds (1 min) length. Signal (MLII). Data standard format. The patient uses this Medication: Digoxin

MIT-BIH Arrhythmia database (mitdb)	109	Male. Age: 64. 60 seconds (1 min) length. Signal (MLII). Data standard format. The patient uses this Medication: Quinidine.

MIT-BIH Arrhythmia database (mitdb)	111	Female. Age: 47. 60 seconds (1 min) length. Signal (MLII). Data standard format. The patient uses these Medications: Digoxin and Lasix.

MIT-BIH Arrhythmia database (mitdb)	114	Female. Age: 72. 60 seconds (1 min) length. Signal (MLII). Data standard format. The patient uses this Medication: Digoxin

MIT-BIH Arrhythmia database (mitdb)	115	Female. Age: 39. 60 seconds (1 min) length. Signal (MLII). Data standard format. The patient does not use any Medication.

MIT-BIH Arrhythmia database (mitdb)	116	Male. Age: 68. 60 seconds (1 min) length. Signal (MLII). Data standard format. The patient does not use any Medication.

MIT-BIH Arrhythmia database (mitdb)	118	Male. Age: 69. 60 seconds (1 min) length. Signal (MLII). Data standard format. The patient uses these Medications: Digoxin and Norpace.
MIT-BIH Arrhythmia database (mitdb)	119	Female. Age: 51. 60 seconds (1 min) length. Signal (MLII). Data standard format. The patient uses this Medication: Pronestyl.

MIT-BIH Arrhythmia database (mitdb)	121	Female. Age: 83. 60 seconds (1 min) length. Signal (MLII). Data standard format. The patient uses these Medications: Digoxin, Isordil and Nitropaste.

Long Term ST database (ltstdb)	s20011	Male. Age: 58. 60 seconds (1 min) length. Signal (ML2). Data standard format. The diagnostic of the patient is: No Coronary artery disease.

Long Term ST database (ltstdb)	s20051	Female. Age: 87. 60 seconds (1 min) length. Signal (ML2). Data standard format. The diagnostic of the patient is: Hypertension.

Long Term ST database (ltstdb)	s20201	Female. Age: 78. 60 seconds (1 min) length. Signal (ML2). Data standard format. The diagnostic of the patient is: Syncope and seizure disorder.

Long Term ST database (ltstdb)	s20272	Male. Age: 61. 60 seconds (1 min) length. Signal (ML2). Data standard format. The diagnostic of the patient is: Coronary artery disease.

The BIDMC Congestive Heart Failure Database	This database includes long-term ECG recordings from 15 subjects (11 men, aged 22 to 71, and 4 women, aged 54 to 63) with severe congestive heart failure (NYHA class 3–4). This group of subjects was part of a larger study group receiving conventional medical therapy *prior* to receiving the oral inotropic agent, milrinone. ECG signals sampled at 250 samples per second with 12-bit resolution over a range of ±10 millivolts.	

The BIDMC Congestive Heart Failure Database	chf01	Gender: not Available Age: Not available. 60 seconds (1 min) length. Signal (ECG1). Data standard format.

The BIDMC Congestive Heart Failure Database	chf02	Gender: not Available Age: Not available. 60 seconds (1 min) length. Signal (ECG1). Data standard format.
The BIDMC Congestive Heart Failure Database	chf03	Gender: not Available Age: Not available. 60 seconds (1 min) length. Signal (ECG1). Data standard format.
The BIDMC Congestive Heart Failure Database	chf04	Gender: not Available Age: Not available. 60 seconds (1 min) length. Signal (ECG1). Data standard format.
The BIDMC Congestive Heart Failure Database	chf05	Gender: not Available Age: Not available. 60 seconds (1 min) length. Signal (ECG1). Data standard format.
The BIDMC Congestive Heart Failure Database	chf06	Gender: not Available Age: Not available. 60 seconds (1 min) length. Signal (ECG1)
The BIDMC Congestive Heart Failure Database	chf07	Gender: not Available Age: Not available. 60 seconds (1 min) length. Signal (ECG1). Data standard format.
The BIDMC Congestive Heart Failure Database	chf08	Gender: not Available Age: Not available. 60 seconds (1 min) length. Signal (ECG1). Data standard format.
The BIDMC Congestive Heart Failure Database	chf09	Gender: not Available Age: Not available. 60 seconds (1 min) length. Signal (ECG1). Data standard format.
The BIDMC Congestive Heart Failure Database	chf10	Gender: not Available Age: Not available. 60 seconds (1 min) length. Signal (ECG1). Data standard format.

**Table 2:** SpO2 database and records that used in simulating algorithms.

Database	Records	Descriptions for all the records
MIMIC Database Numerics	032n, 033n, 037n, 039n, 041n, 048n, 052n, 054n, 055n, 208n, 209n, 210n, 211n, 212n, 213n, 214n, 215n, 216n, 218n, 219n, 220n, 221n, 224n, 225n, 226n, 230n, 231n, 232n, 233n, 235n, 237n, 240n, 241n, 242n, 243n, 245n, 248n, 252n, 253n, 254n, 255n, 259n, 260n, 262n, 264n, 267n, 268n, 224n, 225n and 269n.	60 seconds (1 min) length. Time standard Format. Data standard format. MIMIC Database is called *Numerics* because these measurements are those that typically appear in numeric form on the ICU monitors' screens: heart rate, blood pressure (mean, systolic, diastolic), respiration rate, oxygen saturation, etc. Since the data-gathering protocol was designed to have minimal impact on patient monitoring or care, the selection of measured variables varies among these records, according to the requirements of the ICU staff for appropriate care of the patients in each case. The measurements in these records, sampled at intervals of 1.024 seconds (0.976563 Hz),

**Fig. 2:**
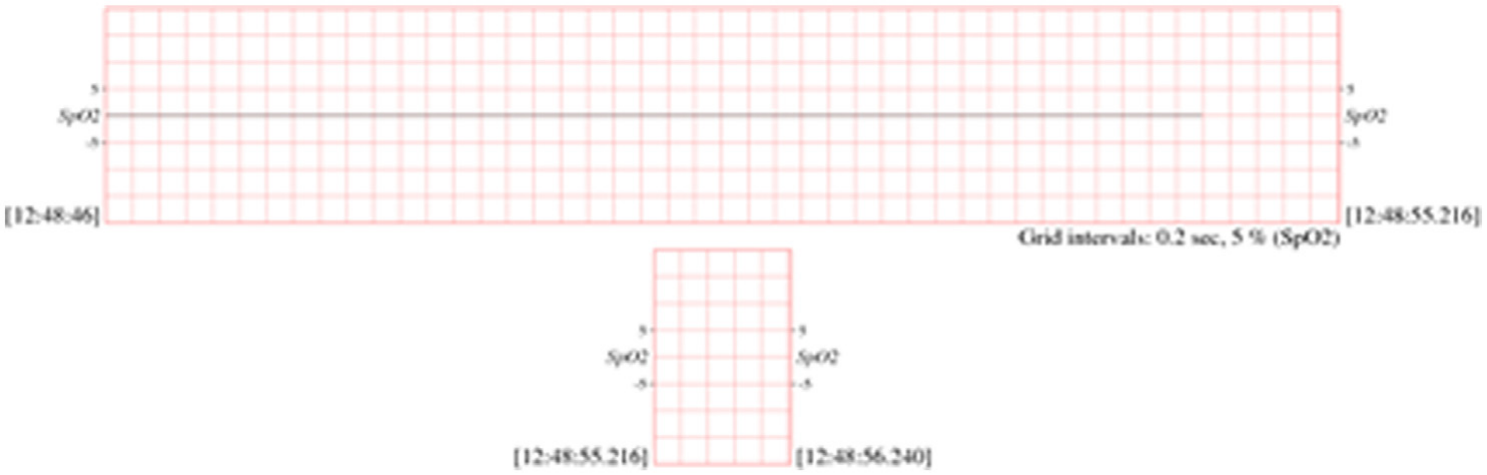
SpO_2_ Sample dataset for record 032n [1].
▪ Table 3 presents the blood pressure signals and database description. A sample of the blood pressure signal is demonstrated in Fig. 3.

**Table 3:** Blood Pressure database and records that used in simulating algorithms dataset for record 032n.

Database	Records	Descriptions for all the records
MIMIC Database Numerics	032n, 033n, 037n, 039n, 041n, 048n, 052n, 054n, 055n, 208n, 209n, 210n, 211n, 212n, 213n, 214n, 215n, 216n, 218n, 219n, 220n, 221n, 224n, 225n, 226n, 230n, 231n, 232n, 233n, 235n, 237n, 240n, 241n, 242n, 243n, 245n, 248n, 252n, 253n, 254n, 255n, 259n, 260n, 262n, 264n, 267n, 268n and 269n.	• 60 seconds (1 min) length. • Time standard Format. • Data standard format. • MIMIC Database is called *Numerics* because these measurements are those that typically appear in numeric form on the ICU monitors' screens: heart rate, blood pressure (mean, systolic, diastolic), respiration rate, oxygen saturation, etc. Since the data-gathering protocol was designed to have minimal impact on patient monitoring or care, the selection of measured variables varies among these records, according to the requirements of the ICU staff for appropriate care of the patients in each case. • The measurements in these records, sampled at intervals of 1.024 seconds (0.976563 Hz),

**Fig. 3:**
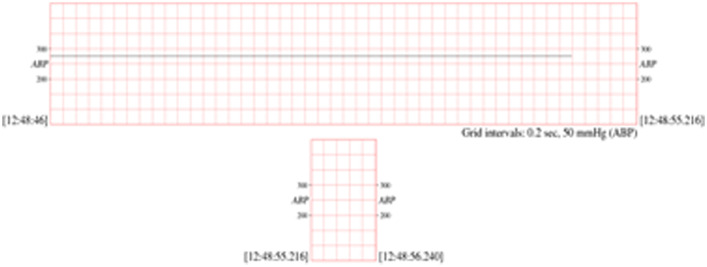
Blood pressure sample dataset for record 032n [1].

These records have been used in our simulation for ECG, blood pressure and SpO2 signals to extract the vital features such as: QRS width; ST elevation; peaks number and cycle interval from ECG signal; SpO2 level from SpO2 signal; high and low blood pressure values from blood pressure signal.

The databases and signal records presented in [1] have been simulated and implemented using our machine learning algorithms. This allows us to extract the essential medical features that are important for healthcare research studies. The outcome of our algorithms is presented in Table 4 as numeric values.

**Table 4:** Vital Features related to blood Pressure diseases extracted based on our simulating algorithms (* x means not addressed).

	Simulated Dataset Descriptions							
Record	Spo2 level	Heart Rates (HR)	Ambulatory Blood PressureSystolic (ABP Sys)	Ambulatory Blood PressureDiastolic (ABP Dias)	Pulmonary Artery Pressure Systolic(PAP Sys)	Pulmonary Artery Pressure Diastolic(PAP Dias)	Non-invasive BP Systolic (NBP Sys)mHg	Non-invasive BP Diastolic (NBP Dias)mHg
032n	0	88	x	x	x	x	x	x
033n	94	53	37	54	x	x	x	x
037n	0	122	82	53	x	x	x	x
039n	98	127	120	73	x	x	x	x
041n	95	88	95	49	27	15	x	x
048n	97	85	164	92	x	x	x	x
052n	99	85	167	78	x	x	x	x
054n	96	93	163	75	x	x	x	x
055n	100	101	114	78	34	22	x	x
208n	93	x	x	x	x	x	116	96
209n	91	x	x	x	x	x	135	65
210n	92	x	x	x	x	x	101	54
221n	97	89	177	63	x	x	x	x
212n	0	80	93	46	29	9	x	x
213n	0	103	111	67	x	x	x	x
214n	93	104	92	55	x	x	150	51
215n	95	71	x	x	78	29	181	62
216n	92	82	113	54	58	26	0	0
218n	94	70	96	57	x	x	0	0
219n	94	73	126	61	x	x	0	0
220n	0	94	141	56	x	x	x	x
221n	99	76	182	80	x	x	x	x
224n	94	x	x	x	x	x	x	x
225n	96	x	x	x	x	x	x	x
226n	97	133	151	81	x	x	x	x
230n	97	72	112	54	59	29	0	0
231n	0	91	122	57	50	25	x	x
232n	97	57	105	63	x	x	x	x
233n	x	74	x	x	x	x	97	57
235n	95	97	154	92	x	x	0	0
237n	97	78	129	76	38	23	0	0
240n	94	81	104	57	39	19	0	0
241n	0	78	140	70	27	36	0	0
242n	99	73	126	68	32	19	0	0
243n	0	112	74	47	35	20	x	x
245n	0	80	105	49	77	35	x	x
248n	94	114	140	60	43	21	x	x
252n	92	60	122	64	x	x	0	0
253n	92	68	129	68	52	25	0	0
254n	96	72	161	93	44	27	0	0
255n	0	97	x	x	30	11	x	x
259n	86	56	102	42	44	20	x	x
260n	0	85	122	35	51	28	0	0
262n	0	70	103	60	41	25	x	x
264n	0	102	0	0	48	17	0	0
267n	100	100	136	64	41	24	0	0
268n	0	99	128	66	63	30	0	0
269n	0	77	117	51	56	28	x	x

Blood pressure and Spo2 datasets provide different values. According to medical guidelines, there are predefined ranges of values that represent the patient condition, which is known as " triage level". This triage level is used to evaluate the performance of the healthcare system, which is specifically focused on patient's medical assessment, e.g., monitoring the patients who have chronic heart diseases or chronic blood pressure diseases. The researchers would need to consider all the probabilities of blood pressure and Spo2 values in their simulation and implementation. Therefore, more analyses would be needed to the dataset records mentioned in [1], which is considered to be time and resources consuming. Hence, the dataset needs to be organized as presented in Table 1, Table 2, Table 3 to allow the researchers to use simplified numeric values in their research work. This essential task has been achieved in our paper so that we have done it on their behalf. Furthermore, we provide the researchers a dataset with different ranges of values that represent different triage levels.

Table 5 demonstrates the dataset with all the probabilities of low blood pressure value, (mHg)high blood pressure value (mHg) and SpO2 value. Moreover, we provide new heterogeneous sources, i.e., text sources. The context of the text-inputs is provided as medical questions. These questions are expressed based on doctors' recommendations. Also, these questions are considered in the doctor diagnostics procedure. The answer to each question considers the feature of each text source. The questions are addressed manually, and all probabilities for the different answers are also considered. These questions can be summarized as follows:

1 Chest pain. The answer is either (Yes) OR (No).
2 Shortness of breath. The answer is either (Yes) OR (No).
3 Palpitation. The answer is either (Yes) OR (No).
4 Patient at rest. The answer is either (Yes) OR (No).

**Table 5:** Vital features related to doctor diagnostics procedure in emergency department and disease detection.

	Patient number						
Simulated Dataset Descriptions	Chest pain	Shortness of Breath	Palpitation.	Patient at rest	SpO2 Value	High Blood Pressure (Bp) Value (mHg)	Low Blood Pressure (Bp) Value (mHg)
1.	N	N	N	N	97	12	23
2.	Y	N	N	N	97	12	23
3.	N	Y	N	N	97	12	23
4.	Y	Y	N	N	97	12	23
5.	N	N	Y	N	97	12	23
6.	Y	N	Y	N	97	12	23
7.	N	Y	Y	N	97	12	23
8.	Y	Y	Y	N	97	12	23
9.	N	N	N	Y	97	12	23
10.	Y	N	N	Y	97	12	23
11.	N	Y	N	Y	97	12	23
12.	Y	Y	N	Y	97	12	23
13.	N	N	Y	Y	97	12	23
14.	Y	N	Y	Y	97	12	23
15.	N	Y	Y	Y	97	12	23
16.	Y	Y	Y	Y	97	12	23
17.	N	N	N	N	92	12	23
18.	Y	N	N	N	92	12	23
19.	N	Y	N	N	92	12	23
20.	Y	Y	N	N	92	12	23
21.	N	N	Y	N	92	12	23
22.	Y	N	Y	N	92	12	23
23.	N	Y	Y	N	92	12	23
24.	Y	Y	Y	N	92	12	23
25.	N	N	N	Y	92	12	23
26.	Y	N	N	Y	92	12	23
27.	N	Y	N	Y	92	12	23
28.	Y	Y	N	Y	92	12	23
29.	N	N	Y	Y	92	12	23
30.	Y	N	Y	Y	92	12	23
31.	N	Y	Y	Y	92	12	23
32.	Y	Y	Y	Y	92	12	23
33.	N	N	N	N	97	10	15
34.	Y	N	N	N	97	10	15
35.	N	Y	N	N	97	10	15
36.	Y	Y	N	N	97	10	15
37.	N	N	Y	N	97	10	15
38.	Y	N	Y	N	97	10	15
39.	N	Y	Y	N	97	10	15
40.	Y	Y	Y	N	97	10	15
41.	N	N	N	Y	97	10	15
42.	Y	N	N	Y	97	10	15
43.	N	Y	N	Y	97	10	15
44.	Y	Y	N	Y	97	10	15
45.	N	N	Y	Y	97	10	15
46.	Y	N	Y	Y	97	10	15
47.	N	Y	Y	Y	97	10	15
48.	Y	Y	Y	Y	97	10	15
49.	N	N	N	N	92	10	15
50.	Y	N	N	N	92	10	15
51.	N	Y	N	N	92	10	15
52.	Y	Y	N	N	92	10	15
53.	N	N	Y	N	92	10	15
54.	Y	N	Y	N	92	10	15
55.	N	Y	Y	N	92	10	15
56.	Y	Y	Y	N	92	10	15
57.	N	N	N	Y	92	10	15
58.	Y	N	N	Y	92	10	15
59.	N	Y	N	Y	92	10	15
60.	Y	Y	N	Y	92	10	15
61.	N	N	Y	Y	92	10	15
62.	Y	N	Y	Y	92	10	15
63.	N	Y	Y	Y	92	10	15
64.	Y	Y	Y	Y	92	10	15
65.	N	N	N	N	97	8	12
66.	Y	N	N	N	97	8	12
67.	N	Y	N	N	97	8	12
68.	Y	Y	N	N	97	8	12
69.	N	N	Y	N	97	8	12
70.	Y	N	Y	N	97	8	12
71.	N	Y	Y	N	97	8	12
72.	Y	Y	Y	N	97	8	12
73.	N	N	N	Y	97	8	12
74.	Y	N	N	Y	97	8	12
75.	N	Y	N	Y	97	8	12
76.	Y	Y	N	Y	97	8	12
77.	N	N	Y	Y	97	8	12
78.	Y	N	Y	Y	97	8	12
79.	N	Y	Y	Y	97	8	12
80.	Y	Y	Y	Y	97	8	12
81.	N	N	N	N	92	8	12
82.	Y	N	N	N	92	8	12
83.	N	Y	N	N	92	8	12
84.	Y	Y	N	N	92	8	12
85.	N	N	Y	N	92	8	12
86.	Y	N	Y	N	92	8	12
87.	N	Y	Y	N	92	8	12
88.	Y	Y	Y	N	92	8	12
89.	N	N	N	Y	92	8	12
90.	Y	N	N	Y	92	8	12
91.	N	Y	N	Y	92	8	12
92.	Y	Y	N	Y	92	8	12
93.	N	N	Y	Y	92	8	12
94.	Y	N	Y	Y	92	8	12
95.	N	Y	Y	Y	92	8	12
96.	Y	Y	Y	Y	92	8	12
97.	N	N	N	N	80	8	12
98.	Y	N	N	N	80	8	12
99.	N	Y	N	N	80	8	12
100.	Y	Y	N	N	80	8	12
101.	N	N	Y	N	80	8	12
102.	Y	N	Y	N	80	8	12
103.	N	Y	Y	N	80	8	12
104.	Y	Y	Y	N	80	8	12
105.	N	N	N	Y	80	8	12
106.	Y	N	N	Y	80	8	12
107.	N	Y	N	Y	80	8	12
108.	Y	Y	N	Y	80	8	12
109.	N	N	Y	Y	80	8	12
110.	Y	N	Y	Y	80	8	12
111.	N	Y	Y	Y	80	8	12
112.	Y	Y	Y	Y	80	8	12
113.	N	N	N	N	80	10	15
114.	Y	N	N	N	80	10	15
115.	N	Y	N	N	80	10	15
116.	Y	Y	N	N	80	10	15
117.	N	N	Y	N	80	10	15
118.	Y	N	Y	N	80	10	15
119.	N	Y	Y	N	80	10	15
120.	Y	Y	Y	N	80	10	15
121.	N	N	N	Y	80	10	15
122.	Y	N	N	Y	80	10	15
123.	N	Y	N	Y	80	10	15
124.	Y	Y	N	Y	80	10	15
125.	N	N	Y	Y	80	10	15
126.	Y	N	Y	Y	80	10	15
127.	N	Y	Y	Y	80	10	15
128.	Y	Y	Y	Y	80	10	15
129.	N	N	N	N	80	12	23
130.	Y	N	N	N	80	12	23
131.	N	Y	N	N	80	12	23
132.	Y	Y	N	N	80	12	23
133.	N	N	Y	N	80	12	23
134.	Y	N	Y	N	80	12	23
135.	N	Y	Y	N	80	12	23
136.	Y	Y	Y	N	80	12	23
137.	N	N	N	Y	80	12	23
138.	Y	N	N	Y	80	12	23
139.	N	Y	N	Y	80	12	23
140.	Y	Y	N	Y	80	12	23
141.	N	N	Y	Y	80	12	23
142.	Y	N	Y	Y	80	12	23
143.	N	Y	Y	Y	80	12	23

According to the medical guidelines, four main ECG features, which are related to many chronic heart diseases, should be extracted. These features are presented as follows:

1 Rhythm, which indicates the sinus bradycardia, sinus tachycardia, atrial tachycardia, atrial flutter, and sick sinus syndrome [9].
2 QRS complex width, which indicates the activity of the bundle branch in the heart [9].
3 Peak-to-peak regularity.
4 ST elevation, which indicates acute myocardial infarction, Prinzmetal's angina, and left ventricular aneurysm [9].

In our evaluation, all the simulated ECG signals represent an abnormal ECG signal. Each signal represents a patient with a certain type of heart disease. We have extracted the four main ECG features and organized them as a new ECG dataset. The researchers can directly use this dataset in their future works. Moreover, to enrich our dataset, we have added our new ECG dataset in Table 5. This dataset becomes easy to access in case the researchers eager to use all the sources in one platform. In addition, we have added our simulation outcomes in terms of triage levels to the table. Our outcomes have already evaluated by medical doctors. Table 6 represents outcomes form our simulation of 580 patients including the formulation of 11 features dataset and variety records of ECG signals where the triage level is provided as output.

**Table 6:** Outcomes form our simulation for 580 patients include formulation of 11 features dataset and variety records of ECG signals and the triage level as output.

p. no.	ECG Records	Spo2	H. Blood(mHg)	L. Blood(mHg)	Chest Pain	Short-ness of Breath	Palpitation.	rest?	Peaks	QRS width	Peak to Peak	ST El.	OutputTriage level
1	Sleep apnea Records	97	23	12	false	false	false	false	67	0.06	Regular	true	Sick
2		97	23	12	false	false	false	true	67	0.06	Regular	true	Sick
3		97	23	12	false	false	true	false	67	0.06	Regular	true	Sick
4		97	23	12	false	false	true	true	67	0.06	Regular	true	Sick
5		97	23	12	false	true	false	false	67	0.06	Regular	true	Sick
6		97	23	12	false	true	false	true	67	0.06	Regular	true	Sick
7		97	23	12	false	true	true	false	67	0.06	Regular	true	Sick
8		97	23	12	false	true	true	true	67	0.06	Regular	true	Urgent
9		97	23	12	true	false	false	false	67	0.06	Regular	true	Sick
10		97	23	12	true	false	false	true	67	0.06	Regular	true	Urgent
11		97	23	12	true	false	true	false	67	0.06	Regular	true	Urgent
12		97	23	12	true	false	true	true	67	0.06	Regular	true	Urgent
13		97	23	12	true	true	false	false	67	0.06	Regular	true	Urgent
14		97	23	12	true	true	false	true	67	0.06	Regular	true	Urgent
15		97	23	12	true	true	true	false	67	0.06	Regular	true	risk
16		97	23	12	true	true	true	true	67	0.06	Regular	true	risk
17		92	23	12	false	false	false	false	67	0.06	Regular	true	Sick
18		92	23	12	false	false	false	true	67	0.06	Regular	true	Sick
19		92	23	12	false	false	true	false	67	0.06	Regular	true	Sick
20		92	23	12	false	false	true	true	67	0.06	Regular	true	Urgent
21		92	23	12	false	true	false	false	67	0.06	Regular	true	Sick
22		92	23	12	false	true	false	true	67	0.06	Regular	true	Sick
23		92	23	12	false	true	true	false	67	0.06	Regular	true	Urgent
24		92	23	12	false	true	true	true	67	0.06	Regular	true	Urgent
25		92	23	12	true	false	false	false	67	0.06	Regular	true	Urgent
26		92	23	12	true	false	false	true	67	0.06	Regular	true	Urgent
27		92	23	12	true	false	true	false	67	0.06	Regular	true	risk
28		92	23	12	true	false	true	true	67	0.06	Regular	true	risk
29		92	23	12	true	true	false	false	67	0.06	Regular	true	Urgent
30		92	23	12	true	true	false	true	67	0.06	Regular	true	Urgent
31		92	23	12	true	true	true	false	67	0.06	Regular	true	risk
32		92	23	12	true	true	true	true	67	0.06	Regular	true	risk
33		97	15	10	false	false	false	false	67	0.06	Regular	true	Sick
34		97	15	10	false	false	false	true	67	0.06	Regular	true	Sick
35		97	15	10	false	false	true	false	67	0.06	Regular	true	Sick
36		97	15	10	false	false	true	true	67	0.06	Regular	true	Sick
37		97	15	10	false	true	false	false	67	0.06	Regular	true	Sick
38		97	15	10	false	true	false	true	67	0.06	Regular	true	Sick
39		97	15	10	false	true	true	false	67	0.06	Regular	true	Sick
40		97	15	10	false	true	true	true	67	0.06	Regular	true	Sick
41		97	15	10	true	false	false	false	67	0.06	Regular	true	Sick
42		97	15	10	true	false	false	true	67	0.06	Regular	true	Sick
43		97	15	10	true	false	true	false	67	0.06	Regular	true	Urgent
44		97	15	10	true	false	true	true	67	0.06	Regular	true	Urgent
45		97	15	10	true	true	false	false	67	0.06	Regular	true	Sick
46		97	15	10	true	true	false	true	67	0.06	Regular	true	Urgent
47		97	15	10	true	true	true	false	67	0.06	Regular	true	Urgent
48		97	15	10	true	true	true	true	67	0.06	Regular	true	Urgent
49		92	15	10	false	false	false	false	67	0.06	Regular	true	Sick
50		92	15	10	false	false	false	true	67	0.06	Regular	true	Sick
51		92	15	10	false	false	true	false	67	0.06	Regular	true	Sick
52		92	15	10	false	false	true	true	67	0.06	Regular	true	Sick
53		92	15	10	false	true	false	false	67	0.06	Regular	true	Sick
54		92	15	10	false	true	false	true	67	0.06	Regular	true	Sick
55		92	15	10	false	true	true	false	67	0.06	Regular	true	Sick
56		92	15	10	false	true	true	true	67	0.06	Regular	true	Urgent
57		92	15	10	true	false	false	false	67	0.06	Regular	true	Sick
58		92	15	10	true	false	false	true	67	0.06	Regular	true	Urgent
59		92	15	10	true	false	true	false	67	0.06	Regular	true	Urgent
60		92	15	10	true	false	true	true	67	0.06	Regular	true	Urgent
61		92	15	10	true	true	false	false	67	0.06	Regular	true	Urgent
62		92	15	10	true	true	false	true	67	0.06	Regular	true	Urgent
63		92	15	10	true	true	true	false	67	0.06	Regular	true	risk
64		92	15	10	true	true	true	true	67	0.06	Regular	true	risk
65		97	12	8	false	false	false	false	67	0.06	Regular	true	Cold State
66		97	12	8	false	false	false	true	67	0.06	Regular	true	Cold State
67		97	12	8	false	false	true	false	67	0.06	Regular	true	Sick
68		97	12	8	false	false	true	true	67	0.06	Regular	true	Sick
69		97	12	8	false	true	false	false	67	0.06	Regular	true	Sick
70		97	12	8	false	true	false	true	67	0.06	Regular	true	Sick
71		97	12	8	false	true	true	false	67	0.06	Regular	true	Sick
72		97	12	8	false	true	true	true	67	0.06	Regular	true	Sick
73		97	12	8	true	false	false	false	67	0.06	Regular	true	Sick
74		97	12	8	true	false	false	true	67	0.06	Regular	true	Sick
75		97	12	8	true	false	true	false	67	0.06	Regular	true	Sick
76		97	12	8	true	false	true	true	67	0.06	Regular	true	Urgent
77		97	12	8	true	true	false	false	67	0.06	Regular	true	Sick
78		97	12	8	true	true	false	true	67	0.06	Regular	true	Sick
79		97	12	8	true	true	true	false	67	0.06	Regular	true	Urgent
80		97	12	8	true	true	true	true	67	0.06	Regular	true	Urgent
81		92	12	8	false	false	false	false	67	0.06	Regular	true	Sick
82		92	12	8	false	false	false	true	67	0.06	Regular	true	Sick
83		92	12	8	false	false	true	false	67	0.06	Regular	true	Sick
84		92	12	8	false	false	true	true	67	0.06	Regular	true	Sick
85		92	12	8	false	true	false	false	67	0.06	Regular	true	Sick
86		92	12	8	false	true	false	true	67	0.06	Regular	true	Sick
87		92	12	8	false	true	true	false	67	0.06	Regular	true	Sick
88		92	12	8	false	true	true	true	67	0.06	Regular	true	Sick
89		92	12	8	true	false	false	false	67	0.06	Regular	true	Sick
90		92	12	8	true	false	false	true	67	0.06	Regular	true	Sick
91		92	12	8	true	false	true	false	67	0.06	Regular	true	Urgent
92		92	12	8	true	false	true	true	67	0.06	Regular	true	Urgent
93		92	12	8	true	true	false	false	67	0.06	Regular	true	Sick
94		92	12	8	true	true	false	true	67	0.06	Regular	true	Urgent
95		92	12	8	true	true	true	false	67	0.06	Regular	true	Urgent
96		92	12	8	true	true	true	true	67	0.06	Regular	true	Urgent
97		80	12	8	false	false	false	false	67	0.06	Regular	true	Sick
98		80	12	8	false	false	false	true	67	0.06	Regular	true	Sick
99		80	12	8	false	false	true	false	67	0.06	Regular	true	Sick
100		80	12	8	false	false	true	true	67	0.06	Regular	true	Sick
101		80	12	8	false	true	false	false	67	0.06	Regular	true	Sick
102		80	12	8	false	true	false	true	67	0.06	Regular	true	Sick
103		80	12	8	false	true	true	false	67	0.06	Regular	true	Sick
104		80	12	8	false	true	true	true	67	0.06	Regular	true	Urgent
105		80	12	8	true	false	false	false	67	0.06	Regular	true	Sick
106		80	12	8	true	false	false	true	67	0.06	Regular	true	Urgent
107		80	12	8	true	false	true	false	67	0.06	Regular	true	Urgent
108		80	12	8	true	false	true	true	67	0.06	Regular	true	Urgent
109		80	12	8	true	true	false	false	67	0.06	Regular	true	Urgent
110		80	12	8	true	true	false	true	67	0.06	Regular	true	Urgent
111		80	12	8	true	true	true	false	67	0.06	Regular	true	risk
112		80	12	8	true	true	true	true	67	0.06	Regular	true	risk
113		80	15	10	false	false	false	false	67	0.06	Regular	true	Sick
114		80	15	10	false	false	false	true	67	0.06	Regular	true	Sick
115		80	15	10	false	false	true	false	67	0.06	Regular	true	Sick
116		80	15	10	false	false	true	true	67	0.06	Regular	true	Urgent
117		80	15	10	false	true	false	false	67	0.06	Regular	true	Sick
118		80	15	10	false	true	false	true	67	0.06	Regular	true	Sick
119		80	15	10	false	true	true	false	67	0.06	Regular	true	Urgent
120		80	15	10	false	true	true	true	67	0.06	Regular	true	Urgent

121	Long Term ST Records	80	15	10	true	false	false	false	67	0.06	Regular	true	Urgent
122		80	15	10	true	false	false	true	67	0.06	Regular	true	Urgent
123		80	15	10	true	false	true	false	67	0.06	Regular	true	risk
124		80	15	10	true	false	true	true	67	0.06	Regular	true	risk
125		80	15	10	true	true	false	false	67	0.06	Regular	true	Urgent
126		80	15	10	true	true	false	true	67	0.06	Regular	true	Urgent
127		80	15	10	true	true	true	false	67	0.06	Regular	true	risk
128		80	15	10	true	true	true	true	67	0.06	Regular	true	risk
129		80	23	12	false	false	false	false	67	0.06	Regular	true	Sick
130		80	23	12	false	false	false	true	67	0.06	Regular	true	Sick
131		80	23	12	false	false	true	false	67	0.06	Regular	true	Urgent
132		80	23	12	false	false	true	true	67	0.06	Regular	true	Urgent
133		80	23	12	false	true	false	false	67	0.06	Regular	true	Sick
134		80	23	12	false	true	false	true	67	0.06	Regular	true	Urgent
135		80	23	12	false	true	true	false	67	0.06	Regular	true	Urgent
136		80	23	12	false	true	true	true	67	0.06	Regular	true	Urgent
137		80	23	12	true	false	false	false	67	0.06	Regular	true	Urgent
138		80	23	12	true	false	false	true	67	0.06	Regular	true	Urgent
139		80	23	12	true	false	true	false	67	0.06	Regular	true	risk
140		80	23	12	true	false	true	true	67	0.06	Regular	true	risk
141		80	23	12	true	true	false	false	67	0.06	Regular	true	risk
142		80	23	12	true	true	false	true	67	0.06	Regular	true	risk
143		80	23	12	true	true	true	false	67	0.06	Regular	true	risk
144		80	23	12	true	true	true	true	67	0.06	Regular	true	risk

145	Arrythmia Records	97	23	12	false	false	false	false	54	0.5	Regular	false	Sick
146		97	23	12	false	false	false	true	54	0.5	Regular	false	Sick
147		97	23	12	false	false	true	false	54	0.5	Regular	false	Sick
148		97	23	12	false	false	true	true	54	0.5	Regular	false	Sick
149		97	23	12	false	true	false	false	54	0.5	Regular	false	Sick
150		97	23	12	false	true	false	true	54	0.5	Regular	false	Sick
151		97	23	12	false	true	true	false	54	0.5	Regular	false	Sick
152		97	23	12	false	true	true	true	54	0.5	Regular	false	Sick
153		97	23	12	true	false	false	false	54	0.5	Regular	false	Sick
154		97	23	12	true	false	false	true	54	0.5	Regular	false	Sick
155		97	23	12	true	false	true	false	54	0.5	Regular	false	Urgent
156		97	23	12	true	false	true	true	54	0.5	Regular	false	Urgent
157		97	23	12	true	true	false	false	54	0.5	Regular	false	Sick
158		97	23	12	true	true	false	true	54	0.5	Regular	false	Urgent
159		97	23	12	true	true	true	false	54	0.5	Regular	false	Urgent
160		97	23	12	true	true	true	true	54	0.5	Regular	false	Urgent
161		92	23	12	false	false	false	false	54	0.5	Regular	false	Sick
162		92	23	12	false	false	false	true	54	0.5	Regular	false	Sick
163		92	23	12	false	false	true	false	54	0.5	Regular	false	Sick
164		92	23	12	false	false	true	true	54	0.5	Regular	false	Sick
165		92	23	12	false	true	false	false	54	0.5	Regular	false	Sick
166		92	23	12	false	true	false	true	54	0.5	Regular	false	Sick
167		92	23	12	false	true	true	false	54	0.5	Regular	false	Sick
168		92	23	12	false	true	true	true	54	0.5	Regular	false	Urgent
169		92	23	12	true	false	false	false	54	0.5	Regular	false	Sick
170		92	23	12	true	false	false	true	54	0.5	Regular	false	Urgent
171		92	23	12	true	false	true	false	54	0.5	Regular	false	Urgent
172		92	23	12	true	false	true	true	54	0.5	Regular	false	Urgent
173		92	23	12	true	true	false	false	54	0.5	Regular	false	Urgent
174		92	23	12	true	true	false	true	54	0.5	Regular	false	Urgent
175		92	23	12	true	true	true	false	54	0.5	Regular	false	risk
176		92	23	12	true	true	true	true	54	0.5	Regular	false	risk
177		97	15	10	false	false	false	false	54	0.5	Regular	false	Cold State
178		97	15	10	false	false	false	true	54	0.5	Regular	false	Cold State
179		97	15	10	false	false	true	false	54	0.5	Regular	false	Sick
180		97	15	10	false	false	true	true	54	0.5	Regular	false	Sick
181		97	15	10	false	true	false	false	54	0.5	Regular	false	Sick
182		97	15	10	false	true	false	true	54	0.5	Regular	false	Sick
183		97	15	10	false	true	true	false	54	0.5	Regular	false	Sick
184		97	15	10	false	true	true	true	54	0.5	Regular	false	Sick
185		97	15	10	true	false	false	false	54	0.5	Regular	false	Sick
186		97	15	10	true	false	false	true	54	0.5	Regular	false	Sick
187		97	15	10	true	false	true	false	54	0.5	Regular	false	Sick
188		97	15	10	true	false	true	true	54	0.5	Regular	false	Urgent
189		97	15	10	true	true	false	false	54	0.5	Regular	false	Sick
190		97	15	10	true	true	false	true	54	0.5	Regular	false	Sick
191		97	15	10	true	true	true	false	54	0.5	Regular	false	Urgent
192		97	15	10	true	true	true	true	54	0.5	Regular	false	Urgent
193		92	15	10	false	false	false	false	54	0.5	Regular	false	Sick
194		92	15	10	false	false	false	true	54	0.5	Regular	false	Sick
195		92	15	10	false	false	true	false	54	0.5	Regular	false	Sick
196		92	15	10	false	false	true	true	54	0.5	Regular	false	Sick
197		92	15	10	false	true	false	false	54	0.5	Regular	false	Sick
198		92	15	10	false	true	false	true	54	0.5	Regular	false	Sick
199		92	15	10	false	true	true	false	54	0.5	Regular	false	Sick
200		92	15	10	false	true	true	true	54	0.5	Regular	false	Sick
201		92	15	10	true	false	false	false	54	0.5	Regular	false	Sick
202		92	15	10	true	false	false	true	54	0.5	Regular	false	Sick
203		92	15	10	true	false	true	false	54	0.5	Regular	false	Urgent
204		92	15	10	true	false	true	true	54	0.5	Regular	false	Urgent
205		92	15	10	true	true	false	false	54	0.5	Regular	false	Sick
206		92	15	10	true	true	false	true	54	0.5	Regular	false	Urgent
207		92	15	10	true	true	true	false	54	0.5	Regular	false	Urgent
208		92	15	10	true	true	true	true	54	0.5	Regular	false	Urgent
209		97	12	8	false	false	false	false	54	0.5	Regular	false	Cold State
210		97	12	8	false	false	false	true	54	0.5	Regular	false	Cold State
211		97	12	8	false	false	true	false	54	0.5	Regular	false	Sick
212		97	12	8	false	false	true	true	54	0.5	Regular	false	Sick
213		97	12	8	false	true	false	false	54	0.5	Regular	false	Cold State
214		97	12	8	false	true	false	true	54	0.5	Regular	false	Cold State
215		97	12	8	false	true	true	false	54	0.5	Regular	false	Sick
216		97	12	8	false	true	true	true	54	0.5	Regular	false	Sick
217		97	12	8	true	false	false	false	54	0.5	Regular	false	Sick
218		97	12	8	true	false	false	true	54	0.5	Regular	false	Sick
219		97	12	8	true	false	true	false	54	0.5	Regular	false	Sick
220		97	12	8	true	false	true	true	54	0.5	Regular	false	Sick
221		97	12	8	true	true	false	false	54	0.5	Regular	false	Sick
222		97	12	8	true	true	false	true	54	0.5	Regular	false	Sick
223		97	12	8	true	true	true	false	54	0.5	Regular	false	Sick
224		97	12	8	true	true	true	true	54	0.5	Regular	false	Urgent
225		92	12	8	false	false	false	false	54	0.5	Regular	false	Cold State
226		92	12	8	false	false	false	true	54	0.5	Regular	false	Cold State
227		92	12	8	false	false	true	false	54	0.5	Regular	false	Sick
228		92	12	8	false	false	true	true	54	0.5	Regular	false	Sick
229		92	12	8	false	true	false	false	54	0.5	Regular	false	Sick
230		92	12	8	false	true	false	true	54	0.5	Regular	false	Sick
231		92	12	8	false	true	true	false	54	0.5	Regular	false	Sick
232		92	12	8	false	true	true	true	54	0.5	Regular	false	Sick
233		92	12	8	true	false	false	false	54	0.5	Regular	false	Sick
234		92	12	8	true	false	false	true	54	0.5	Regular	false	Sick
235		92	12	8	true	false	true	false	54	0.5	Regular	false	Sick
236		92	12	8	true	false	true	true	54	0.5	Regular	false	Urgent
237		92	12	8	true	true	false	false	54	0.5	Regular	false	Sick
238		92	12	8	true	true	false	true	54	0.5	Regular	false	Sick
239		92	12	8	true	true	true	false	54	0.5	Regular	false	Urgent
240		92	12	8	true	true	true	true	54	0.5	Regular	false	Urgent
241		80	12	8	false	false	false	false	54	0.5	Regular	false	Sick
242		80	12	8	false	false	false	true	54	0.5	Regular	false	Sick
243		80	12	8	false	false	true	false	54	0.5	Regular	false	Sick
244		80	12	8	false	false	true	true	54	0.5	Regular	false	Sick
245		80	12	8	false	true	false	false	54	0.5	Regular	false	Sick
246		80	12	8	false	true	false	true	54	0.5	Regular	false	Sick
247		80	12	8	false	true	true	false	54	0.5	Regular	false	Sick
248		80	12	8	false	true	true	true	54	0.5	Regular	false	Sick
249		80	12	8	true	false	false	false	54	0.5	Regular	false	Sick
250		80	12	8	true	false	false	true	54	0.5	Regular	false	Sick
251		80	12	8	true	false	true	false	54	0.5	Regular	false	Urgent
252		80	12	8	true	false	true	true	54	0.5	Regular	false	Urgent
253		80	12	8	true	true	false	false	54	0.5	Regular	false	Sick
254		80	12	8	true	true	false	true	54	0.5	Regular	false	Urgent
255		80	12	8	true	true	true	false	54	0.5	Regular	false	Urgent
256		80	12	8	true	true	true	true	54	0.5	Regular	false	Urgent
257		80	15	10	false	false	false	false	54	0.5	Regular	false	Sick
258		80	15	10	false	false	false	true	54	0.5	Regular	false	Sick
259		80	15	10	false	false	true	false	54	0.5	Regular	false	Sick
260		80	15	10	false	false	true	true	54	0.5	Regular	false	Sick
261		80	15	10	false	true	false	false	54	0.5	Regular	false	Sick
262		80	15	10	false	true	false	true	54	0.5	Regular	false	Sick
263		80	15	10	false	true	true	false	54	0.5	Regular	false	Sick
264		80	15	10	false	true	true	true	54	0.5	Regular	false	Urgent
265		80	15	10	true	false	false	false	54	0.5	Regular	false	Sick
266		80	15	10	true	false	false	true	54	0.5	Regular	false	Urgent
267		80	15	10	true	false	true	false	54	0.5	Regular	false	Urgent
268		80	15	10	true	false	true	true	54	0.5	Regular	false	Urgent
269		80	15	10	true	true	false	false	54	0.5	Regular	false	Urgent
270		80	15	10	true	true	false	true	54	0.5	Regular	false	Urgent
271		80	15	10	true	true	true	false	54	0.5	Regular	false	risk
272		80	15	10	true	true	true	true	54	0.5	Regular	false	risk
273		80	23	12	false	false	false	false	54	0.5	Regular	false	Sick
274		80	23	12	false	false	false	true	54	0.5	Regular	false	Sick
275		80	23	12	false	false	true	false	54	0.5	Regular	false	Sick
276		80	23	12	false	false	true	true	54	0.5	Regular	false	Urgent
277		80	23	12	false	true	false	false	54	0.5	Regular	false	Sick
278		80	23	12	false	true	false	true	54	0.5	Regular	false	Sick
279		80	23	12	false	true	true	false	54	0.5	Regular	false	Urgent
280		80	23	12	false	true	true	true	54	0.5	Regular	false	Urgent
281		80	23	12	true	false	false	false	54	0.5	Regular	false	Urgent
282		80	23	12	true	false	false	true	54	0.5	Regular	false	Urgent
283		80	23	12	true	false	true	false	54	0.5	Regular	false	risk
284		80	23	12	true	false	true	true	54	0.5	Regular	false	risk
285		80	23	12	true	true	false	false	54	0.5	Regular	false	Urgent
286		80	23	12	true	true	false	true	54	0.5	Regular	false	Urgent
287		80	23	12	true	true	true	false	54	0.5	Regular	false	risk
288		80	23	12	true	true	true	true	54	0.5	Regular	false	risk
289		97	23	12	false	false	false	false	77	0.047	Regular	true	Sick
290		97	23	12	false	false	false	true	77	0.047	Regular	true	Sick
291		97	23	12	false	false	true	false	77	0.047	Regular	true	Sick
292		97	23	12	false	false	true	true	77	0.047	Regular	true	Sick
293		97	23	12	false	true	false	false	77	0.047	Regular	true	Sick
294		97	23	12	false	true	false	true	77	0.047	Regular	true	Sick
295		97	23	12	false	true	true	false	77	0.047	Regular	true	Urgent
296		97	23	12	false	true	true	true	77	0.047	Regular	true	Urgent
297		97	23	12	true	false	false	false	77	0.047	Regular	true	Urgent
298		97	23	12	true	false	false	true	77	0.047	Regular	true	Urgent
299		97	23	12	true	false	true	false	77	0.047	Regular	true	risk
300		97	23	12	true	false	true	true	77	0.047	Regular	true	risk
301		97	23	12	true	true	false	false	77	0.047	Regular	true	Urgent
302		97	23	12	true	true	false	true	77	0.047	Regular	true	Urgent
303		97	23	12	true	true	true	false	77	0.047	Regular	true	risk
304		97	23	12	true	true	true	true	77	0.047	Regular	true	risk
305		92	23	12	false	false	false	false	77	0.047	Regular	true	Sick
306		92	23	12	false	false	false	true	77	0.047	Regular	true	Sick
307		92	23	12	false	false	true	false	77	0.047	Regular	true	Urgent
308		92	23	12	false	false	true	true	77	0.047	Regular	true	Urgent
309		92	23	12	false	true	false	false	77	0.047	Regular	true	Sick
310		92	23	12	false	true	false	true	77	0.047	Regular	true	Sick
311		92	23	12	false	true	true	false	77	0.047	Regular	true	Urgent
312		92	23	12	false	true	true	true	77	0.047	Regular	true	Urgent
313		92	23	12	true	false	false	false	77	0.047	Regular	true	Urgent
314		92	23	12	true	false	false	true	77	0.047	Regular	true	Urgent
315		92	23	12	true	false	true	false	77	0.047	Regular	true	risk
316		92	23	12	true	false	true	true	77	0.047	Regular	true	risk
317		92	23	12	true	true	false	false	77	0.047	Regular	true	risk
318		92	23	12	true	true	false	true	77	0.047	Regular	true	risk
319		92	23	12	true	true	true	false	77	0.047	Regular	true	risk
320		92	23	12	true	true	true	true	77	0.047	Regular	true	risk
321		97	15	10	false	false	false	false	77	0.047	Regular	true	Sick
322		97	15	10	false	false	false	true	77	0.047	Regular	true	Sick
323		97	15	10	false	false	true	false	77	0.047	Regular	true	Sick
324		97	15	10	false	false	true	true	77	0.047	Regular	true	Sick
325		97	15	10	false	true	false	false	77	0.047	Regular	true	Sick
326		97	15	10	false	true	false	true	77	0.047	Regular	true	Sick
327		97	15	10	false	true	true	false	77	0.047	Regular	true	Sick
328		97	15	10	false	true	true	true	77	0.047	Regular	true	Sick
329		97	15	10	true	false	false	false	77	0.047	Regular	true	Sick
330		97	15	10	true	false	false	true	77	0.047	Regular	true	Sick
331		97	15	10	true	false	true	false	77	0.047	Regular	true	Urgent
332		97	15	10	true	false	true	true	77	0.047	Regular	true	Urgent
333		97	15	10	true	true	false	false	77	0.047	Regular	true	Urgent
334		97	15	10	true	true	false	true	77	0.047	Regular	true	Urgent
335		97	15	10	true	true	true	false	77	0.047	Regular	true	risk
336		97	15	10	true	true	true	true	77	0.047	Regular	true	risk
337		92	15	10	false	false	false	false	77	0.047	Regular	true	Sick
338		92	15	10	false	false	false	true	77	0.047	Regular	true	Sick
339		92	15	10	false	false	true	false	77	0.047	Regular	true	Sick
340		92	15	10	false	false	true	true	77	0.047	Regular	true	Sick
341		92	15	10	false	true	false	false	77	0.047	Regular	true	Sick
342		92	15	10	false	true	false	true	77	0.047	Regular	true	Sick
343		92	15	10	false	true	true	false	77	0.047	Regular	true	Urgent
344		92	15	10	false	true	true	true	77	0.047	Regular	true	Urgent
345		92	15	10	true	false	false	false	77	0.047	Regular	true	Urgent
346		92	15	10	true	false	false	true	77	0.047	Regular	true	Urgent
347		92	15	10	true	false	true	false	77	0.047	Regular	true	risk
348		92	15	10	true	false	true	true	77	0.047	Regular	true	risk
349		92	15	10	true	true	false	false	77	0.047	Regular	true	Urgent
350		92	15	10	true	true	false	true	77	0.047	Regular	true	Urgent
351		92	15	10	true	true	true	false	77	0.047	Regular	true	risk
352		92	15	10	true	true	true	true	77	0.047	Regular	true	risk
353		97	12	8	false	false	false	false	77	0.047	Regular	true	Cold State
354		97	12	8	false	false	false	true	77	0.047	Regular	true	Sick
355		97	12	8	false	false	true	false	77	0.047	Regular	true	Sick
356		97	12	8	false	false	true	true	77	0.047	Regular	true	Sick
357		97	12	8	false	true	false	false	77	0.047	Regular	true	Sick
358		97	12	8	false	true	false	true	77	0.047	Regular	true	Sick
359		97	12	8	false	true	true	false	77	0.047	Regular	true	Sick
360		97	12	8	false	true	true	true	77	0.047	Regular	true	Sick
361		97	12	8	true	false	false	false	77	0.047	Regular	true	Sick
362		97	12	8	true	false	false	true	77	0.047	Regular	true	Sick
363		97	12	8	true	false	true	false	77	0.047	Regular	true	Urgent
364		97	12	8	true	false	true	true	77	0.047	Regular	true	Urgent
365		97	12	8	true	true	false	false	77	0.047	Regular	true	Sick
366		97	12	8	true	true	false	true	77	0.047	Regular	true	Sick
367		97	12	8	true	true	true	false	77	0.047	Regular	true	Urgent
368		97	12	8	true	true	true	true	77	0.047	Regular	true	Urgent
369		92	12	8	false	false	false	false	77	0.047	Regular	true	Sick
370		92	12	8	false	false	false	true	77	0.047	Regular	true	Sick
371		92	12	8	false	false	true	false	77	0.047	Regular	true	Sick
372		92	12	8	false	false	true	true	77	0.047	Regular	true	Sick
373		92	12	8	false	true	false	false	77	0.047	Regular	true	Sick
374		92	12	8	false	true	false	true	77	0.047	Regular	true	Sick
375		92	12	8	false	true	true	false	77	0.047	Regular	true	Sick
376		92	12	8	false	true	true	true	77	0.047	Regular	true	Sick
377		92	12	8	true	false	false	false	77	0.047	Regular	true	Sick
378		92	12	8	true	false	false	true	77	0.047	Regular	true	Sick
379		92	12	8	true	false	true	false	77	0.047	Regular	true	Urgent
380		92	12	8	true	false	true	true	77	0.047	Regular	true	Urgent
381		92	12	8	true	true	false	false	77	0.047	Regular	true	Urgent
382		92	12	8	true	true	false	true	77	0.047	Regular	true	Urgent
383		92	12	8	true	true	true	false	77	0.047	Regular	true	risk
384		92	12	8	true	true	true	true	77	0.047	Regular	true	risk
385		80	12	8	false	false	false	false	77	0.047	Regular	true	Sick
386		80	12	8	false	false	false	true	77	0.047	Regular	true	Sick
387		80	12	8	false	false	true	false	77	0.047	Regular	true	Sick
388		80	12	8	false	false	true	true	77	0.047	Regular	true	Sick
389		80	12	8	false	true	false	false	77	0.047	Regular	true	Sick
390		80	12	8	false	true	false	true	77	0.047	Regular	true	Sick
391		80	12	8	false	true	true	false	77	0.047	Regular	true	Urgent
392		80	12	8	false	true	true	true	77	0.047	Regular	true	Urgent
393		80	12	8	true	false	false	false	77	0.047	Regular	true	Urgent
394		80	12	8	true	false	false	true	77	0.047	Regular	true	Urgent
395		80	12	8	true	false	true	false	77	0.047	Regular	true	risk
396		80	12	8	true	false	true	true	77	0.047	Regular	true	risk
397		80	12	8	true	true	false	false	77	0.047	Regular	true	Urgent
398		80	12	8	true	true	false	true	77	0.047	Regular	true	Urgent
399		80	12	8	true	true	true	false	77	0.047	Regular	true	risk
400		80	12	8	true	true	true	true	77	0.047	Regular	true	risk
401		80	15	10	false	false	false	false	77	0.047	Regular	true	Sick
402		80	15	10	false	false	false	true	77	0.047	Regular	true	Sick
403		80	15	10	false	false	true	false	77	0.047	Regular	true	Urgent
404		80	15	10	false	false	true	true	77	0.047	Regular	true	Urgent
405		80	15	10	false	true	false	false	77	0.047	Regular	true	Sick
406		80	15	10	false	true	false	true	77	0.047	Regular	true	Sick
407		80	15	10	false	true	true	false	77	0.047	Regular	true	Urgent
408		80	15	10	false	true	true	true	77	0.047	Regular	true	Urgent
409		80	15	10	true	false	false	false	77	0.047	Regular	true	Urgent
410		80	15	10	true	false	false	true	77	0.047	Regular	true	Urgent
411		80	15	10	true	false	true	false	77	0.047	Regular	true	risk
412		80	15	10	true	false	true	true	77	0.047	Regular	true	risk
413		80	15	10	true	true	false	false	77	0.047	Regular	true	risk
414		80	15	10	true	true	false	true	77	0.047	Regular	true	risk
415		80	15	10	true	true	true	false	77	0.047	Regular	true	risk
416		80	15	10	true	true	true	true	77	0.047	Regular	true	risk
417		80	23	12	false	false	false	false	77	0.047	Regular	true	Sick
418		80	23	12	false	false	false	true	77	0.047	Regular	true	Sick
419		80	23	12	false	false	true	false	77	0.047	Regular	true	Urgent
420		80	23	12	false	false	true	true	77	0.047	Regular	true	Urgent
421		80	23	12	false	true	false	false	77	0.047	Regular	true	Urgent
422		80	23	12	false	true	false	true	77	0.047	Regular	true	Urgent
423		80	23	12	false	true	true	false	77	0.047	Regular	true	risk
424		80	23	12	false	true	true	true	77	0.047	Regular	true	risk
425		80	23	12	true	false	false	false	77	0.047	Regular	true	risk
426		80	23	12	true	false	false	true	77	0.047	Regular	true	risk
427		80	23	12	true	false	true	false	77	0.047	Regular	true	risk
428		80	23	12	true	false	true	true	77	0.047	Regular	true	risk
429		80	23	12	true	true	false	false	77	0.047	Regular	true	risk
430		80	23	12	true	true	false	true	77	0.047	Regular	true	risk
431		80	23	12	true	true	true	false	77	0.047	Regular	true	risk
432		80	23	12	true	true	true	true	77	0.047	Regular	true	risk

433	Heart failure records	97	23	12	false	false	false	false	64	0.169	Regular	false	Cold State
434		97	23	12	false	false	false	true	64	0.169	Regular	false	Cold State
435		97	23	12	false	false	true	false	64	0.169	Regular	false	Sick
436		97	23	12	false	false	true	true	64	0.169	Regular	false	Sick
437		97	23	12	false	true	false	false	64	0.169	Regular	false	Sick
438		97	23	12	false	true	false	true	64	0.169	Regular	false	Sick
439		97	23	12	false	true	true	false	64	0.169	Regular	false	Sick
440		97	23	12	false	true	true	true	64	0.169	Regular	false	Sick
441		97	23	12	true	false	false	false	64	0.169	Regular	false	Sick
442		97	23	12	true	false	false	true	64	0.169	Regular	false	Sick
443		97	23	12	true	false	true	false	64	0.169	Regular	false	Sick
444		97	23	12	true	false	true	true	64	0.169	Regular	false	Urgent
445		97	23	12	true	true	false	false	64	0.169	Regular	false	Sick
446		97	23	12	true	true	false	true	64	0.169	Regular	false	Sick
447		97	23	12	true	true	true	false	64	0.169	Regular	false	Urgent
448		97	23	12	true	true	true	true	64	0.169	Regular	false	Urgent
449		92	23	12	false	false	false	false	64	0.169	Regular	false	Sick
450		92	23	12	false	false	false	true	64	0.169	Regular	false	Sick
451		92	23	12	false	false	true	false	64	0.169	Regular	false	Sick
452		92	23	12	false	false	true	true	64	0.169	Regular	false	Sick
453		92	23	12	false	true	false	false	64	0.169	Regular	false	Sick
454		92	23	12	false	true	false	true	64	0.169	Regular	false	Sick
455		92	23	12	false	true	true	false	64	0.169	Regular	false	Sick
456		92	23	12	false	true	true	true	64	0.169	Regular	false	Sick
457		92	23	12	true	false	false	false	64	0.169	Regular	false	Sick
458		92	23	12	true	false	false	true	64	0.169	Regular	false	Sick
459		92	23	12	true	false	true	false	64	0.169	Regular	false	Urgent
460		92	23	12	true	false	true	true	64	0.169	Regular	false	Urgent
461		92	23	12	true	true	false	false	64	0.169	Regular	false	Sick
462		92	23	12	true	true	false	true	64	0.169	Regular	false	Urgent
463		92	23	12	true	true	true	false	64	0.169	Regular	false	Urgent
464		92	23	12	true	true	true	true	64	0.169	Regular	false	Urgent
465		97	15	10	false	false	false	false	64	0.169	Regular	false	Cold State
466		97	15	10	false	false	false	true	64	0.169	Regular	false	Cold State
467		97	15	10	false	false	true	false	64	0.169	Regular	false	Sick
468		97	15	10	false	false	true	true	64	0.169	Regular	false	Sick
469		97	15	10	false	true	false	false	64	0.169	Regular	false	Cold State
470		97	15	10	false	true	false	true	64	0.169	Regular	false	Cold State
471		97	15	10	false	true	true	false	64	0.169	Regular	false	Sick
472		97	15	10	false	true	true	true	64	0.169	Regular	false	Sick
473		97	15	10	true	false	false	false	64	0.169	Regular	false	Sick
474		97	15	10	true	false	false	true	64	0.169	Regular	false	Sick
475		97	15	10	true	false	true	false	64	0.169	Regular	false	Sick
476		97	15	10	true	false	true	true	64	0.169	Regular	false	Sick
477		97	15	10	true	true	false	false	64	0.169	Regular	false	Sick
478		97	15	10	true	true	false	true	64	0.169	Regular	false	Sick
479		97	15	10	true	true	true	false	64	0.169	Regular	false	Sick
480		97	15	10	true	true	true	true	64	0.169	Regular	false	Urgent
481		92	15	10	false	false	false	false	64	0.169	Regular	false	Cold State
482		92	15	10	false	false	false	true	64	0.169	Regular	false	Cold State
483		92	15	10	false	false	true	false	64	0.169	Regular	false	Sick
484		92	15	10	false	false	true	true	64	0.169	Regular	false	Sick
485		92	15	10	false	true	false	false	64	0.169	Regular	false	Sick
486		92	15	10	false	true	false	true	64	0.169	Regular	false	Sick
487		92	15	10	false	true	true	false	64	0.169	Regular	false	Sick
488		92	15	10	false	true	true	true	64	0.169	Regular	false	Sick
489		92	15	10	true	false	false	false	64	0.169	Regular	false	Sick
490		92	15	10	true	false	false	true	64	0.169	Regular	false	Sick
491		92	15	10	true	false	true	false	64	0.169	Regular	false	Sick
492		92	15	10	true	false	true	true	64	0.169	Regular	false	Urgent
493		92	15	10	true	true	false	false	64	0.169	Regular	false	Sick
494		92	15	10	true	true	false	true	64	0.169	Regular	false	Sick
495		92	15	10	true	true	true	false	64	0.169	Regular	false	Urgent
496		92	15	10	true	true	true	true	64	0.169	Regular	false	Urgent
497		97	12	8	false	false	false	false	64	0.169	Regular	false	Cold State
498		97	12	8	false	false	false	true	64	0.169	Regular	false	Cold State
499		97	12	8	false	false	true	false	64	0.169	Regular	false	Cold State
500		97	12	8	false	false	true	true	64	0.169	Regular	false	Cold State

501	Normal ECG	97	23	12	Y	Y	N	N	normal		Regular		Sick
502		97	23	12	Y	Y	N	Y	normal		Regular		Sick
503		97	23	12	Y	Y	Y	N	normal		Regular		Sick
504		97	23	12	Y	Y	Y	Y	normal		Regular		Sick
505		97	15	10	Y	Y	N	N	normal		Regular		Sick
506		97	15	10	Y	Y	N	Y	normal		Regular		Sick
507		97	15	10	Y	Y	Y	N	normal		Regular		Sick
508		97	15	10	Y	Y	Y	Y	normal		Regular		Sick
509		97	12	8	Y	Y	N	N	normal		Regular		Cold State
510		97	12	8	Y	Y	N	Y	normal		Regular		Sick
511		97	12	8	Y	Y	Y	N	normal		Regular		Sick
512		97	12	8	Y	Y	Y	Y	normal		Regular		Sick
513		97	12	8	N	N	Y	Y	normal		Regular		Cold State
514		97	12	8	N	N	Y	N	normal		Regular		Cold State
515		97	12	8	N	N	N	Y	normal		Regular		Normal
516		97	12	8	N	N	N	N	normal		Regular		Normal
517		97	12	8	Y	N	Y	Y	normal		Regular		Sick
518		97	12	8	Y	N	Y	N	normal		Regular		Sick
519		97	12	8	Y	N	N	Y	normal		Regular		Cold State
520		97	12	8	Y	N	N	N	normal		Regular		Cold State
521		97	12	8	N	Y	Y	Y	normal		Regular		Cold State
522		97	12	8	N	Y	N	Y	normal		Regular		Cold State
523		97	12	8	N	Y	Y	N	normal		Regular		Cold State
524		97	12	8	N	Y	N	N	normal		Regular		Cold State
525		97	15	8	Y	N	Y	Y	normal		Regular		Sick
526		97	15	8	Y	N	Y	N	normal		Regular		Sick
527		97	15	8	Y	N	N	Y	normal		Regular		Cold State
528		97	15	8	Y	N	N	N	normal		Regular		Cold State
529		97	15	8	N	Y	Y	Y	normal		Regular		Cold State
530		97	15	8	N	Y	N	Y	normal		Regular		Cold State
531		97	15	8	N	Y	Y	N	normal		Regular		Cold State
532		97	15	8	N	Y	N	N	normal		Regular		Cold State
533		97	12	10	Y	N	Y	Y	normal		Regular		Sick
534		97	12	10	Y	N	Y	N	normal		Regular		Sick
535		97	12	10	Y	N	N	Y	normal		Regular		Cold State
536		97	12	10	Y	N	N	N	normal		Regular		Cold State
537		97	12	10	Y	Y	Y	Y	normal		Regular		Sick
538		97	12	10	Y	Y	Y	N	normal		Regular		Sick
539		97	12	10	Y	Y	N	Y	normal		Regular		Cold State
540		97	12	10	Y	Y	N	N	normal		Regular		Cold State
541		97	12	10	N	Y	Y	Y	normal		Regular		Cold State
542		97	12	10	N	Y	N	Y	normal		Regular		Cold State
543		97	12	10	N	Y	Y	N	normal		Regular		Cold State
544		97	12	10	N	Y	N	N	normal		Regular		Cold State
545		97	12	8	Y	N	Y	Y	normal		Regular		Sick
546		97	12	8	Y	N	Y	N	normal		Regular		Sick
547		97	12	8	Y	N	N	Y	normal		Regular		Cold State
548		97	12	8	Y	N	N	N	normal		Regular		Cold State
549		97	12	8	Y	Y	Y	Y	normal		Regular		Sick
550		97	12	8	Y	Y	Y	N	normal		Regular		Sick
551		97	12	8	Y	Y	N	Y	normal		Regular		Cold State
552		97	12	8	Y	Y	N	N	normal		Regular		Cold State
553	Normal ECG with HR 110	97	12	8	N	N	N	Y	110		Regular		Normal
554		97	12	8	N	N	N	Y	110		Regular		Normal
555		97	12	8	N	N	N	Y	110		Regular		Normal
556		97	12	8	N	N	N	Y	110		Regular		Normal
557		97	12	8	N	N	N	Y	110		Regular		Normal
558		97	12	8	N	N	N	Y	110		Regular		Normal
559		97	12	8	N	N	N	Y	110		Regular		Normal
560		97	12	8	N	N	N	Y	110		Regular		Normal
561		97	12	8	N	N	N	Y	110		Regular		Normal
562		97	12	8	N	N	N	Y	110		Regular		Normal
563		97	12	8	N	N	N	Y	110		Regular		Normal
564		97	12	8	N	N	N	Y	110		Regular		Normal
565		97	12	8	N	N	N	Y	110		Regular		Normal
566		97	12	8	N	N	N	Y	110		Regular		Normal
567		97	12	8	N	N	N	Y	110		Regular		Normal
568		97	12	8	N	N	N	Y	110		Regular		Normal
569		97	12	8	N	N	N	Y	110		Regular		Normal
570		97	12	8	N	N	N	Y	110		Regular		Normal
571		97	12	8	N	N	N	Y	110		Regular		Normal
572		97	12	8	N	N	N	Y	110		Regular		Normal
573		92	12	8	N	N	N	Y	110		Regular		Sick
574		92	12	8	N	N	N	Y	110		Regular		Sick
575		92	12	8	N	N	N	Y	110		Regular		Sick
576		92	12	8	N	N	N	Y	110		Regular		Sick
577		92	12	8	N	N	N	Y	110		Regular		Sick
578		92	12	8	N	N	N	Y	110		Regular		Sick
579		92	12	8	N	N	N	Y	110		Regular		Sick
580		92	12	8	N	N	N	Y	110		Regular		Sick

Table 7 presents dataset used in our paper [7] to provide different packages of healthcare services in the telemedicine environment.

**Table 7:** formulation of 10 patients’ dataset for evaluating healthcare services provisioning system in telemedicine environment.

Patient Alias Name	ECGrecord name	spo2 record	Blood Pressure record (High)	Blood Pressure record (Low)	Patient index in MSHA simulation	Spo2 value	High Blood Pressure value (mHg)	Low Blood Pressure value (mHg)	Location	Chest Pain	Shortness of Breath.	Palpitations	rest?	ECG Number of Peaks	QRS width	P-P Interval	ST Elevation
Mrs. smith	a01	259n	032n	209n	144	80	23	12	Home	true	true	true	true	67	0.06	0.065763	true
Ross	105	052n	048n	414n	363	97	12	8	Home	true	false	true	false	77	0.047	0.266642	true
Monica	103	0481n	032n	209n	160	97	23	12	Home	true	true	true	true	54	0.5	0.037372	false
Joey	105	210n	048n	33n	342	92	15	10	Home	false	true	false	true	77	0.047	0.266642	true
Sally	107	217n	032n	209n	436	97	23	12	Home	false	false	true	true	64	0.169	0.336318	false
James	normal	048n	219n	219n	65	97	12	8	Home	false	false	false	false	67	0.06	0.065763	true
Rayan	normal	052n	048n	414n	66	97	12	8	Home	false	false	false	true	67	0.06	0.065763	true
Chander	normal	054n	219n	219n	515	97	12	8	Home	N	N	N	Y	normal			
Sarah	normal	048n	048n	414n	516	97	12	8	Home	N	N	N	N	normal			
Pheby	normal	052n	219n	219n	553	97	12	8	Home	N	N	N	Y	110	normal		

## 2 Experimental design, materials and methods

### 2.1 Simulation setup

The software architecture of our algorithms is implemented using JAVA programming language. This is because JAVA has many benefits, such as: (a) real-time implementation, (b) parallel execution, (c) usage from anywhere by all interested parties, (d) ability to run JAVA-based applications on different platforms, (e) and compatibility to be used with different operating systems, e.g., Android, Windows, and Linux. The advantages of using JAVA have paved the way for the implementation of our algorithms in different hardware platforms. XAMMP has also been used. Specifically, XAMPP is a small and light Apache distribution tool that contains the most common web development technologies in a single package. XAMPP is a free/open-source software, and its name stands for (X) cross-platform for Web server, HTTP Apache Server, (M) MySQL database, (P) PHP scripts writing language, and (P) Perl programming language. In our paper, the dataset is re-organized and re-formatted in structure dataset format. The dataset is represented in terms of tables to illustrate the patient's profile and the type of diseases.

### 2.2 Computational analytic methods and codes

To extract the dataset mentioned in Table 4, Table 5, Table 6, advanced processing algorithms have been applied to the signals mentioned in Table 1, Table 2, Table 3. To this end, a multi-function data processing algorithm is proposed and implemented [7] in order to extract the essential features from each source individually. Each signal is represented by an array.

According to the extracted dataset, each element in the signal has two values. The first value represents time and the second represents voltage. The array of each signal has two columns (each column represents a value). The number of rows is defined by the number of elements in the signal, which starts from (0) and ends at (n). The array of text feature is 1 × 4 because there are four variables that represent four features. A real-time data processing algorithm have been utilized to extract ECG features. The ECG signal is represented by an array of two columns (time in (ms) and voltage in (mv)). These values have been used to extract the features. The ECG signal provides many cycles. One ECG cycle has many ECG features such as: Rythem; QRS; ST; and P-P.

For each cycle, the signal values in time are varied around the zero lines. These values are used to split the ECG cycle to Up and Down halves, then sorting the upper half based on voltage values. This is then applied to find the maximum point, which is represented by the R point. Accordingly, the upper half of the ECG cycle can be splatted into right and half. As such, by using certain functions to sort the values of the ECG cycle for each half (Up_Lift and Up_right) based on (t) value and (v) value, the location of Q and S points can be found. Moreover, the ST elevation can be determined based on the differences of (t) and (v) values using the subtraction functions. The SpO2 and blood pressure values have been calculated as mentioned in [6].

The proposed algorithm is presented as pseudo-codes to enable the researchers to implement it in any software platform. Moreover, the algorithm is implemented using Java code, which is provided in the attached appendix.

## 3 Ethics statement

The authors would like to point out that the primary data sources are available in a public repository and given in PhysioNet online library [1]. PhysioNet online library includes many types of medical raw datasets. PhysioNet online library gives the permission to all researchers around the world to download and use the raw datasets. However, our main contribution is presented in applying signal processing algorithms in order to extract the essential vital features from the raw datasets. Consequently, the essential raw dataset and the outcomes of the simulated data are organized, structured, formulated and presented as multi diseases dataset.

Finally, the authors would like to indicate that neither human subjects nor animal experiments are involved in this paper.

## CRediT Author Statement

**Omar H. Salman:** Responsible for methodology, conceptualization, designing the algorithms, simulation and writing the original draft. **Mohammed I. Aal-Nouman**: his task was visualization and investigation the state-of-the-art related research works. **Zahraa K. Taha**: Responsible for software development, data curation, and writing the article. **Muntadher Q. Alsabah:** Responsible for proofreading the paper and improve the English writing of our manuscript. **Yaseein S. Hussein:** his task was to review the paper and provide some useful comments regarding the paper organization and development. **Zahraa Adnan:** Responsible for reviewing the dataset tables, gathering related information, and providing technical comments regarding the features’ extraction.

## Declaration of Competing Interest

The authors would like to declare that there are no competing financial interests or personal relationships which have, or could be perceived to have, influenced the work presented in this paper.
